# Polymyxin B hemoperfusion: a mechanistic perspective

**DOI:** 10.1186/cc13912

**Published:** 2014-06-09

**Authors:** Claudio Ronco, David J Klein

**Affiliations:** 1 Department of Nephrology, Dialysis and Transplantation and International Renal Research Institute (IRRIV), San Bortolo Hospital, Vicenza 36100, Italy; 2 Department of Critical Care, and the Keenan Research Centre, Li Ka Shing Knowledge Institute, St Michael’s Hospital, University of Toronto, Toronto M5B 1W8, Canada

## Abstract

Direct hemoperfusion therapy with polymyxin B immobilized fiber cartridge (PMX-DHP) is an established strategy in the treatment of septic shock in Japan and parts of Western Europe. PMX-DHP is currently the subject of a pivotal North American randomized controlled trial (EUPHRATES) in patients with septic shock and confirmed endotoxemia, as measured by the endotoxin activity assay. The major mechanism of action of this therapy is the removal of circulating endotoxin. High affinity binding of circulating endotoxin by the PMX-DHP column may decrease circulating endotoxin levels by up to 90% after two standard treatments. Basic research has shown reductions in circulating cytokine levels and in renal tubular apoptosis. Clinical research has shown that PMX-DHP therapy results in hemodynamic improvements, improvements in oxygenation, renal function, and reductions in mortality. Further research is needed to further define additional patient populations with endotoxemia that may benefit from PMX-DHP therapy as well as to further elucidate dosing, timing, and additional information on mechanisms of action. This review will present the mechanistic rationale for this targeted strategy of endotoxin removal using PMX-DHP in endotoxemic septic patients, highlighting both the specific effects of the therapy and the evidence accumulated so far of clinical improvement following this therapy in terms of recovery of organ function.

## Targeting lipopolysaccharide in septic shock

This viewpoint discusses the proven and potential mechanisms of action for polymyxin B hemoperfusion (PMX-DHP) as a biologically plausible and clinically efficacious therapy in sepsis and endotoxemia. The role of endotoxin or lipopolysaccharide (LPS) in sepsis is well established with over 10,000 publications in the medical literature. LPS is a key component of the membrane of Gram-negative bacteria. Structurally, its biologic activity relates to the lipid A portion of the molecule, which is highly conserved across most bacterial species [1]. LPS when injected systemically in both animals and humans in a dose-dependent fashion induces elevations in cytokines, including TNF-α, IL-6, and IL-8 [2,3]. Clinical responses include pyrexia, chills, hypotension, and, at higher doses, shock, organ failure and death [4]. Numerous studies have shown elevated levels of circulating LPS in patients with sepsis originating not only from Gram-negative infections, but also in patients with Gram-positive infections, and in those where cultures do not identify a microbiologic source of sepsis [5,6]. The likely mechanism of endotoxemia in these cases is the translocation of gut microbial flora secondary to intestinal hypoperfusion. Animal studies supporting this hypothesis have included those that have identified high levels of circulating LPS after supra-celiac aortic cross clamping for aortic surgery [7,8]. Similar observations of endotoxemia have been noted in human cardiopulmonary bypass, severe burn, liver transplant, and trauma patients [9,10].

The possibility to do these studies has been bolstered by the development of the endotoxin activity assay (EAA), which has allowed accurate measurement of endotoxin activity levels *in vivo*[11]. The EAA measures the activity of circulating bio-available lipid A, thus overcoming many of the interference issues that exist with the limulus ameobocyte lysate assay. Elevations in the EAA correlate well with the observed clinical status of patients. EAA levels in sepsis patients in the ICU correlate with adverse outcomes, including risk of death and duration of hospital stay [5]. It has also been demonstrated that elevation in the EAA can provide additional prognostic value in assessing patients with milder or earlier forms of sepsis, in perioperative major surgery patients, and in severe sepsis and septic shock patients for whom specific anti-endotoxin therapies are being contemplated [12-14].

**Figure 1:**
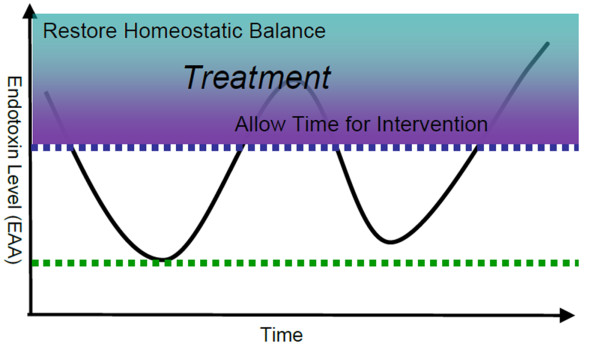
**Time frame of events and windows of opportunity for treatment.** EAA, endotoxin activity assay.

Importantly, studies have demonstrated persistent elevations in LPS levels as measured by the EAA up to 3 days after initial development of severe sepsis. Furthermore, the total exposure to circulating endotoxin as measured by the area under the curve when EAA levels are plotted over the first 3 days of admission (endotoxin burden) correlates with the degree of total organ failure [15]. Organ failure is the biggest correlate to attributable mortality in sepsis [16]. Finally, fluctuations in circulating endotoxin levels in septic patients have been shown to be associated with adverse outcomes [15]. This has led to the hypothesis that both the elimination of high levels of circulating endotoxin and the blunting of the changes in these levels over time is a potential therapeutic strategy in endotoxemia with associated septic shock (Figure 1).

## Polymyxin B

Polymyxin B is a cyclic cationic polypeptide antibiotic derived from *Bacillus polymyxa* that has the ability to bind and neutralize endotoxin [17]. Studies have shown that systemic polymyxin B blunts the TNF-α response to endotoxin [18]. Polymyxin B also blocks the formation of LPS-LPS binding protein complexes through its high binding affinity for the LPS molecule. Unfortunately, infusion of polymyxin in humans results in nephrotoxicity and neurotoxicity, limiting its intravenous use to salvage therapy for Gram-negative enterobacteriaciae resistant to other antibiotics [17]. Clinical trials of polymyxin B as an anti-sepsis/anti-endotoxin agent bound to dextran failed to advance through clinical trials in the 1990s, again related to systemic toxicity [19].

## Hemoperfusion with polymyxin B immobilized cartridges

**Figure 2:**
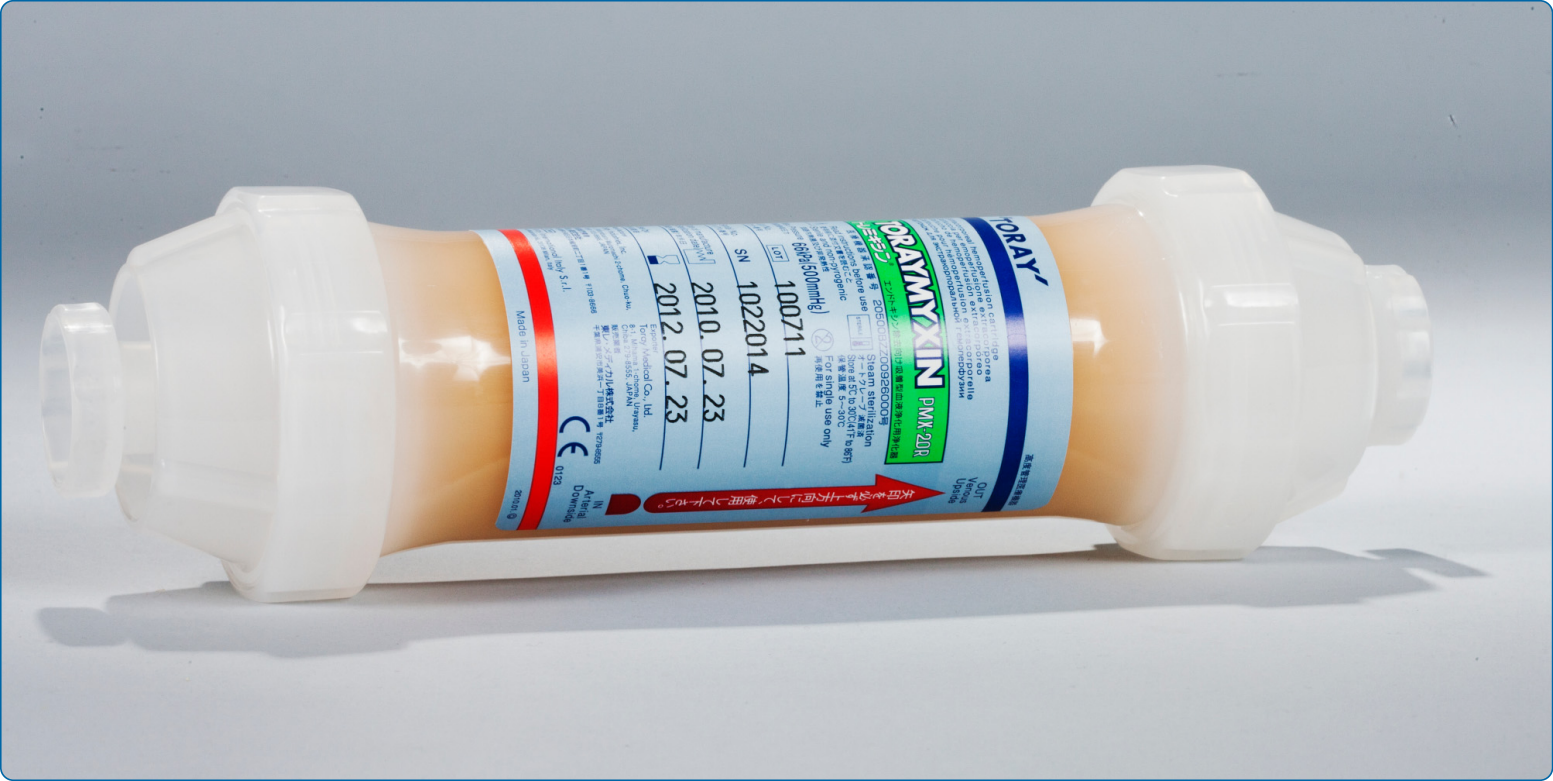
Polymixin B cartridge containing polystyrenic fibers bound to polymixin-B.

A novel therapeutic strategy whereby polymyxin B is immobilized to a polystyrene-derived fiber in a hemoperfusion device that is used to remove circulating endotoxin was developed in Japan in the early 1990s [20] (Figure 2). The PMX cartridge was created by covalently immobilizing polymyxin B to polystyrene-derived fibers, which can then be used to filter blood externally using an extracorporeal circuit, thereby removing circulating LPS through its adsorption to the PMX cartridge. The surface area of the cartridge is extremely large, allowing up to 90% of circulating endotoxin to be cleared in a short period of time, as demonstrated by Shoji and colleagues [20]. Clinically, the EAA-J study confirmed that endotoxin levels may fluctuate in the inter-treatment period thought to be related to the movement of endotoxin out of other compartments (protein bound, micelles, ongoing septic sources), reinforcing the potential rationale for current practice of two treatments 24 hours apart [21]. To date, PMX-DHP has been used in more than 100,000 patients with a very low incidence of adverse events (<1%) and high tolerability. The most commonly observed adverse events are thrombocytopenia, transient hypotension and allergic reactions. These have been logged by regulators in North America, Europe, and Japan, as well as in the EUPHAS2 registry currently active in Europe, and in published literature. There is no evidence that polymyxin B enters the systemic circulation in significant quantities in treated patients.

Small randomized studies on this device have also shown the ability of the PMX cartridge to dramatically reduce endotoxin concentrations compared with carrier fiber cartridges, charcoal or resin over the course of a 2-hour treatment [22]. Due to the strong binding capacity of polymyxin B and the affinity of polymyxin B for the highly conserved lipid A portion of LPS, the device has also been shown to remove a large variety of the various endotoxins produced by an array of different Gram-negative bacteria, showing the versatility of this treatment. Recently, Tani and colleagues [23] tested the endotoxin adsorption capacity of the device, reporting that the PMX cartridge is able to trap approximately 300,000 endotoxin units in a standard 2-hour perfusion session. This correlates well with the observed order of magnitude of the typical endotoxin burden during severe sepsis. Similarly, the EAA-J trial demonstrated an average absolute 20% reduction in EAA levels (from 0.65 to 0.45) in an unselected cohort of septic patients who received PMX-DHP on clinical grounds at the discretion of the treating physician, representing very roughly a 2,000 pg/ml reduction in endotoxin levels [21].

## Mechanism of action

**Figure 3:**
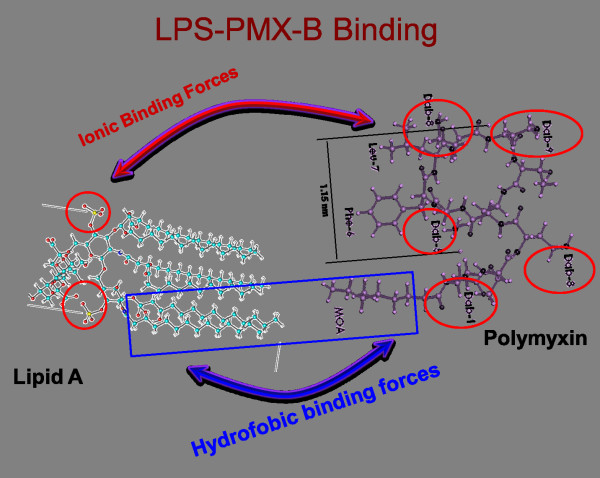
**Lipopolysaccharide (LPS) binds to polymixin B (PMX) with weak ionic forces and strong hydrophobic forces.** This differentiates this type of removal from any other system. Dab, hydrophilic residues; Leu, Leucine; MOA, methyl octanoic acid; Phe, Phenylalanine.

The main mechanism of action behind PMX-DHP is the direct adsorption of circulating LPS. At the molecular level this relies on both the polystyrene-polymyxin B bonding and the LPS-polymyxin B affinity (Figure 3). The first bond is the covalent linkage of polymyxin B to the fiber surface, thus protecting the patient from the nephrotoxic and neurotoxic effects of polymyxin B. Molecular studies on polymyxin B and LPS interaction have determined that a stable bond is created, with hydrophobic interactions dominating the interaction for intermolecular distances up to 1 nm, such as between the lipid A and polymyxin B hydrophobic residues. At larger intermolecular distances, LPS phosphate groups, with negatives charges, interact with positively charged diaminobutyric acid residues of polymyxin B, giving rise to ionic interactions [24]. The large surface area of the PMX column exerts fluid drag but allows for a large amount of interaction with the fiber. Fiore and colleagues [25] have shown that, within a wide range of wall sheer stresses, the capability of polymyxin B to capture endotoxin molecules extends to distances of one order of magnitude greater than the characteristic distance of the stable intermolecular hydrophobic bond. Therefore, the polymyxin adsorbed to the fiber is able to capture LPS from a threshold distance of 10 to 20 nm during the normal blood flow of a standard treatment.

**Figure 4:**
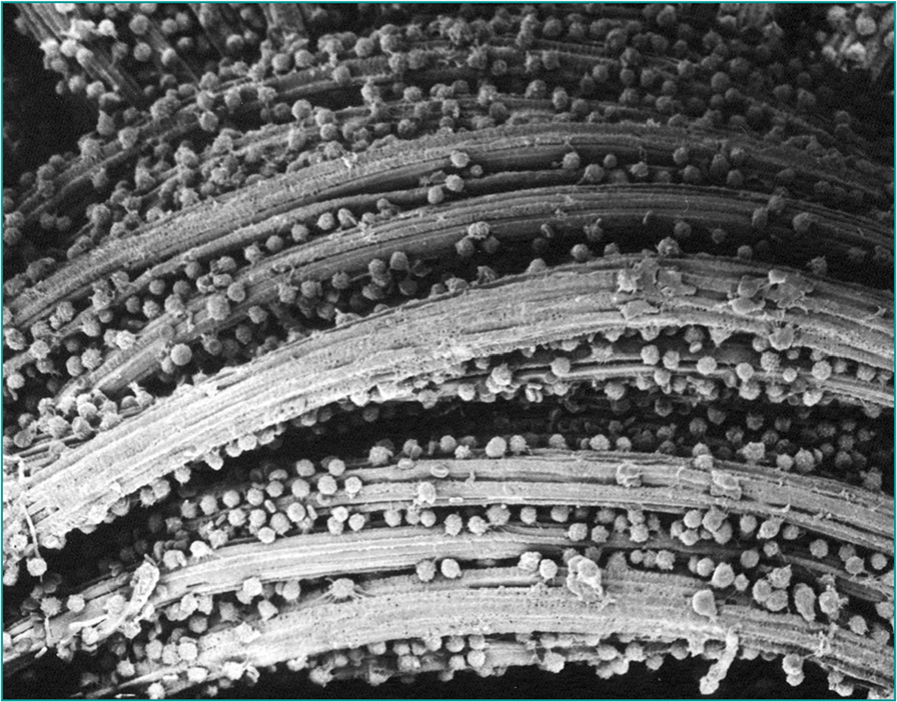
Pictorial view of activated cells adsorbed by the fibers.

As a secondary mechanism the PMX column has also been reported to remove inflammatory cells such as monocytes and neutrophils [26]. While it is unlikely that these inflammatory cells are removed through binding with PMX directly, they may be removed by either being physically entrapped by the fiber weave of the filter or attaching to LPS, which in turn binds to polymyxin, thereby using LPS as an adsorption bridge (Figure 4). White cell count reductions have been observed clinically in some patients treated with PMX-DHP. In either case, the removal of activated inflammatory cells can have the secondary effect of reducing the circulating levels of inflammatory mediators, such as the cytokines TNF-α and IL-6. Consequently, the exaggerated systemic immune response of patients can be reduced, preventing or decreasing multiorgan failure.

Interestingly, PMX-DHP treatment has also been associated with decreasing circulating apoptotic factors, such as Fas, Fas-L, Bax, Bcl-2, and reductions in the levels of renal tubular and glomerular cellular apoptosis, which in turn was associated with recovery of renal tubular formation, reduced inflammation and improved renal function [27]. This provides another potential mechanism involved in the overall reduction in organ dysfunction following PMX-DHP, including a rationale for the often observed clinical effect on acute kidney injury.

There are other reports of PMX-DHP therapy changing levels of other measurable players in the inflammatory response in sepsis. These include lipotichoeic acid, HMGB-1, and even mediators of the inflammatory response in amiodarone toxicity [28,29]. However, there is little causal evidence to support a direct role for PMX in these cases. The mechanisms for this are unclear and may reflect pleiotropic effects of reducing endotoxin burden.

## Animal data

The first large mammal safety study of PMX was done in 20 dogs infused with *Escherichia coli* in a Gram-negative sepsis model. It demonstrated the ability of PMX-DHP to reduce circulating endotoxin levels, improve blood pressure, and reduce mortality in treated animals [30]. Further studies in guinea pigs and sheep have noted improved gastrointestinal perfusion and reductions in clinical parameters of shock and hypoxemia in sepsis models [31,32]. Recently, animal studies from Japan have focused on potential new areas for clinical use through studies on a rat sepsis model with endotoxin-induced acute lung injury/acute respiratory distress syndrome [33].

## Clinical data

### Small studies

There are many published trials of small and somewhat heterogeneous cohorts of patients mainly from Japan as well as case reports and case series from elsewhere demonstrating impressive efficacy of PMX-DHP in severe sepsis. Studies on human subjects have been reported since 1994 [34], with more than 120 English language publications reporting on over 2,000 patients treated with PMX-DHP now in the public domain. The first multi-center trial was done by Tani and colleagues in 1998 [35], where 37 of 70 subjects with severe sepsis were treated with PMX-DHP and the treatment group demonstrated an 18% reduction in absolute 14-day mortality compared to the control population.

### Meta-analyses/systematic reviews

Recently, Zhou and collaborators [36] published a meta-analysis of all randomized trials of blood purification strategies in sepsis, including high volume hemofiltration, PMX-DHP, and plasma exchange. Importantly, while they showed an overall reduction in mortality from 50.1% to 35.7% (odds ratio 0.69, 95% confidence interval 0.56 to 0.84), they highlighted that the result would no longer be significant if the eight trials on PMX-DHP with 457 patients were excluded, thus suggesting that the clinical effect that drove the result of the meta-analysis was principally due to the PMX-DHP trials.

Cruz and colleagues published an earlier systematic review and meta-analysis of 28 trials where PMX-DHP was used to treat patients with severe sepsis and septic shock. Although there was heterogeneity amongst the trials, which were largely done in Europe and Japan, across a total cohort of 978 patients treated with the therapy, improvements were noted in hemodynamics (mean arterial pressure) as well as oxygenation (P/F ratio), and there was a statistically significant improvement in risk of death (risk ratio 0.53, 95% confidence interval 0.43 to 0.65) [37]. Nevertheless, these data need to be considered as hypothesis generating, since despite the strong positive clinical signal, the included trials are largely underpowered, unblinded, and have variable inclusion criteria.

### Randomized controlled trials

#### European pilot trial

A European multicenter pilot trial was conducted by Vincent and colleagues [38] in six academic centers in France, Belgium, Spain, Germany, The Netherlands and the UK. The study was powered for safety and surrogate non-mortality endpoints. Treatment consisted of only a single PMX cartridge, which is considered half of the current recommended dose. Thirty-six septic patients were randomized to PMX- DHP (n = 17) or standard care (n = 19). There was no difference in mortality, although there was a statistically significant improvement in mean arterial pressure and other hemodynamic measures as well as a reduction in the need for renal replacement therapy in the treated group. From the safety perspective, authors reported a safety profile that was favorable towards PMX-DHP versus the control population.

#### EUPHAS

In 2009, the Early Use of Polymyxin Hemoperfusion in Abdominal Septic Shock (EUPHAS) trial, a randomized unblinded study of 64 patients in 10 tertiary Italian ICUs, demonstrated statistically significant improvements in the primary endpoints of hemodynamics and organ dysfunction. Specifically, renal function as measured by improvement of serum creatinine, diuresis, and the renal component of the Sequential Organ Failure Assessment score at 72 hours showed positive trends. Also, the absolute risk of death at 28 days improved significantly from 53% in the conventional therapy group to 32% in the PMX-DHP treated group (*P* = 0.03). These results, albeit encouraging, are considered controversial as the trial was stopped early after an interim analysis showed a mortality difference, which was a secondary endpoint. In addition, the trial was not blinded. Patients were selected for therapy based on evidence of septic shock from an intra-abdominal source to hopefully enrich the patient population with likely endotoxemic patients, but the EAA was not measured. Despite these limitations, EUPHAS may have had a comparative advantage to other sepsis trials that selected patients for anti-endotoxin therapy regardless of source of sepsis or measured levels of endotoxin.

### Ongoing studies

#### EUPHAS2

EUPHAS2 is a prospective web-based registry of patients treated with PMX-DHP designed to validate the reproducibility of randomized clinical trial results and to observe the 'real world' efficacy and utility of PMX-DHP therapy across a wider variety of patient populations than those included in clinical trials. To date, the registry has captured data on 426 treated patients. Unpublished data from the registry that have been presented to date seem to show similar benefits to those observed in EUPHAS and other trials.

#### ABDO-MIX

The effects of Hemoperfusion With a Polymyxin B Membrane in Peritonitis With Septic Shock (ABDO-MIX; ClinicalTrials.gov NCT01222663) is a French randomized, controlled, open label multi-centered study that evaluated 28 day mortality in patients with septic shock due to peritonitis. Eligible patients were randomized to standard care versus standard care plus PMX-DHP within 36 hours of abdominal surgery to repair a hollow viscous perforation. The trial enrolled 240 patients. Although this study has not been published, preliminary results were presented at the 2014 ISICEM conference. This study failed to show a difference in mortality between the groups, but the study had a number of potential problems that may have contributed to this observation. These include cartridge clotting and failure rates that are dramatically higher than in other trials or clinical experience to date suggesting technical issues in the implementation of the therapy protocol. Also, the observed composite mortality was substantially less than the estimate included in the sample size calculation, thus decreasing the power of the study to detect a difference. Still, once published, ABDO-MIX should be carefully reviewed in the context of other previous and emerging data.

#### EUPHRATES

The EUPHRATES trial (Evaluating the Use of Polymyxin B Hemoperfusion in a Randomized Controlled Trial of Adults Treated for Endotoxemia and Septic Shock; ClinicalTrials.Gov NCT01046669) is a multi-centered, blinded, randomized controlled trial of PMX-DHP in patients with septic shock and confirmed endotoxemia using EAA greater than 0.60. The trial is being conducted in 50 ICUs in the United States and Canada and aims to enroll 360 patients, but has an adaptive design that allows for a sample size adjustment based on a Data and Safety Monitoring Board interim analysis. The primary endpoint for the trial is 28-day all-cause mortality. Unique features of the trial include absence of systemic inflammatory response syndrome criteria as a requirement for inclusion, use of the EAA to confirm endotoxemia as a requisite for treatment, and use of a detailed 'fascade' hemoperfusion event as a blinding mechanism. Similar to previous studies, PMX-DHP is being done in two treatment sessions of 2 hours duration approximately 1 day apart. Patients are enrolled after persistent septic shock despite adequate fluid resuscitation who remain on vaspressors for at least 2 but not more than 30 hours and have an EAA >0.60.

## Conclusion

PMX-DHP is a well-tolerated and safe treatment for septic shock with a long history of clinical experience and both clinical and basic science data to support efficacy in endotoxemia. Its principle mechanism of action is through the removal of circulating endotoxin, although its effects are likely pleiotropic. In an era of numerous failed clinical trials in sepsis, it is easy to be cynical. However, this personalized, targeted approach to a disease with unacceptable mortality, with a treatment with a long history of clinical use and strong supporters around the globe truly may represent a step forward in improving patient care.

## Abbreviations

EAA: Endotoxin activity assay; EUPHAS: Early use of polymyxin hemoperfusion in abdominal septic shock; IL: Interleukin; LPS: Lipopolysaccharide; PMX-DHP: Polymyxin B hemoperfusion; TNF: Tumor necrosis factor.

## Competing interests

CR has received honoraria as a member of the steering committee of the EUPHAS2 registry. DJK has served as a consultant to Spectral Diagnostics Inc., the sponsor of the EUPHRATES trial, and serves as the medical monitor for the trial.

## References

[B1] Danner RL Elin RJ Hosseini JM Wesley RA Reilly JM Parillo JE Endotoxemia in human septic shock Chest 1991 99 169 175 10.1378/chest.99.1.169 1984950

[B2] Casey LC Balk RA Bone RC Plasma cytokine and endotoxin levels correlate with survival in patients with the sepsis syndrome Ann Intern Med 1993 119 771 778 10.7326/0003-4819-119-8-199310150-00001 8379598

[B3] Hoffman WD Natanson C Endotoxin in septic shock Anesth Analg 1993 77 613 624 8368563 10.1213/00000539-199309000-00032

[B4] Burrell R Human responses to bacterial endotoxin Circ Shock 1994 43 137 153 7850934

[B5] Marshall JC Foster D Vincent JL Cook DJ Cohen J Dellinger RP Opal S Abraham E Brett SJ Smith T Mehta S Derzko A Romaschin A MEDIC study Diagnostic and prognostic implications of endotoxemia in critical illness: results of the MEDIC study J Infect Dis 2004 190 527 534 10.1086/422254 15243928

[B6] Opal SM Cohen J Clinical gram-positive sepsis: does it fundamentally differ from gram-negative bacterial sepsis? Crit Care Med 1999 27 1608 1616 10.1097/00003246-199908000-00039 10470773

[B7] Kim OY Monsel A Bertrand M Cavaillon JM Coriat P Adib-Conquy M Translocation of bacterial NOD2 agonist and its link with inflammation Crit Care 2009 13 R124 10.1186/cc7980 19638210 PMC2750177

[B8] Bahrami S Schlag G Yao YM Redl H Significance of translocation/endotoxin in the development of systemic sepsis following trauma and/or haemorrhage Prog Clin Biol Res 1995 392 197 208 8524925

[B9] Klein DJ Briet F Nisenbaum R Romaschin AD Mazer CD Endotoxemia related to cardiopulmonary bypass is associated with increased risk of infection after cardiac surgery: a prospective observational study Crit Care 2011 15 R69 10.1186/cc10051 21345192 PMC3222002

[B10] DeGasperi A DeCian W Vaiani F Corti A Sabbadini D Pannacciulli E Amici O Mazza E Cristalli A Prosperi M Fantini G Notaro P Vai S Scailoa A Santandrea E Endotoxemia following liver transplantation in humans Transplant Proc 1994 26 3664 3665 7998312

[B11] Marshall JC Walker PM Foster DM Harris D Ribeiro M Paice J Romaschin AD Derzko AN Measurement of endotoxin activity in critically ill patients using whole blood neutrophil dependent chemiluminescence Crit Care 2002 6 342 348 10.1186/cc1522 12225611 PMC125316

[B12] Biagioni E Venturelli C Klein DJ Buoncristiano M Rumpianesi F Busani S Rinaldi L Donati A Girardis M Endotoxin activity levels as a prediction tool for risk of deterioration in patients with sepsis not admitted to the intensive care unit: a pilot observational study J Crit Care 2012 28 612 617 23602034 10.1016/j.jcrc.2013.02.005

[B13] Yaguchi A Yuzawa J Klein DJ Takeda M Harada T Combining intermediate levels of the Endotoxin Activity Assay (EAA) with other biomarkers in the assessment of patients with sepsis: results of an observational study Crit Care 2013 16 R88 22607642 10.1186/cc11350 PMC3580633

[B14] Novelli G Ferretti G Ruberto F Morabito V Pugliese F Early management of endotoxemia using the endotoxin activity assay and polymyxin B-based hemoperfusion Contrib Nephrol 2010 167 91 101 20519903 10.1159/000315923

[B15] Klein DJ Derzko A Foster D Seely AJ Brunet F Romaschin AD Marshall JC Daily variation in endotoxin levels is associated with increased organ failure in critically ill patients Shock 2007 28 524 529 17589381 10.1097/shk.0b013e31805363c6

[B16] Levy MM Macias WL Vincent JL Russell JA Silva E Trzaskoma B Williams MD Early changes in organ function predict eventual survival in severe sepsis Crit Care Med 2005 33 2194 2201 10.1097/01.CCM.0000182798.39709.84 16215369

[B17] Zavascki AP Goldani LZ Li J Nation RL Polymyxin B for the treatment of multidrug-resistant pathogens: a critical review J Antimicrob Chemother 2007 60 1206 1215 10.1093/jac/dkm357 17878146

[B18] Sharp CR DeClue AE Haak CE Honaker AR Reinero CR Evaluation of the anti-endotoxin effects of polymyxin B in a feline model of endotoxemia J Feline Med Surg 2010 12 278 285 10.1016/j.jfms.2009.12.014 20156699 PMC11135593

[B19] Opal SM Gluck T Endotoxin as a drug target Crit Care Med 2003 31 S57 S64 10.1097/00003246-200301001-00009 12544978

[B20] Shoji H Tani T Hanasawa K Kodama M Extracorporeal endotoxin removal by polymyxin B immobilized fiber cartridge: designing and antiendotoxin efficacy in the clinical application Ther Apher 1998 2 3 12 10.1111/j.1744-9987.1998.tb00066.x 10227782

[B21] Romaschin AD Klein DJ Marshall JC Bench-to-bedside review: Clinical experience with the endotoxin activity assay Crit Care 2012 16 248 10.1186/cc11495 23206992 PMC3672550

[B22] Ronco C Piccinni P Kellum J Rationale of extracorporeal removal endotoxin in sepsis: theory, timing and technique Contrib Nephrol 2010 167 25 34 20519896 10.1159/000315916

[B23] Tani T Shoji H Guadagni G Perego A Extracorporeal removal of endotoxin: the polymyxin B-immobilized fiber cartridge Contrib Nephrol 2010 167 35 44 20519897 10.1159/000315917

[B24] Vesentini S Soncini M Zaupa A Silvestri V Fiore GB Redaelli A Multi-scale analysis of the toraymyxin adsorption cartridge. Part I: molecular interaction of polymyxin B with endotoxins Int J Artif Organs 2006 29 239 250 16552671 10.1177/039139880602900210

[B25] Fiore B Soncini M Vesentini S Penati A Visconti G Redaelli A Multi-scale analysis of the toraymyxin adsorption cartridge. Part II: computational fluid-dynamic study Int J Artif Organs 2006 29 251 260 16552672 10.1177/039139880602900211

[B26] Nishibori M Takahashi HK Katayama H Mori S Saito S Iwagaki H Tanaka N Morita K Ohtsuka A Specific removal of monocytes from peripheral blood of septic patients by polymyxin B-immobilized filter column Acta Med Okayama 2009 63 65 69 19247424 10.18926/AMO/31855

[B27] Cantaluppi V Assenzio B Pasero D Romanazzi GM Pacitti A Lanfranco G Puntorieri V Martin EL Mascia L Monti G Casella G Segoloni GP Camussi G Ranieri VM Polymyxin-B hemoperfusion inactivates circulating proapoptotic factors Intensive Care Med 2008 34 1638 1645 10.1007/s00134-008-1124-6 18463848 PMC2517091

[B28] Jaber BL Barrett TW Cendoroglo Neto M Sundaram S King AJ Pereira BJ Removal of cytokine inducing substances by polymyxin-B immobilized polystyrene-derivative fibers during in vitro hemoperfusion of 10% human plasma containing Staphylococcus aureus challenge ASAIO J 1998 44 48 53 10.1097/00002480-199801000-00011 9466501

[B29] Sato N Kojima K Horio Y Goto E Masunaga A Ichiyasu H Kohrogi H Successful treatment of severe amiodarone pulmonary toxicity with polymyxin B-immobilized fiber column direct hemoperfusion Chest 2013 143 1146 1150 10.1378/chest.12-0994 23546489

[B30] Sato T Orlowski JP Zborowski M Experimental study of extracorporeal perfusion for septic shock ASAIO J 1993 39 M790 M793 8268646

[B31] Yamamoto H Koizumi T Kaneki T Fujimoto K Kubo K Honda T Direct hemoperfusion with polymyxin B-immobilized fiber improves shock and hypoxemia during endotoxemia in anesthetized sheep J Endotoxin Res 2002 8 419 426 10.1177/09680519020080061001 12542853

[B32] Nakanowatari Y Nemoto K Hara S Ninomiya N Yamamoto Y Effects of direct haemoperfusion through fibres immobilizing polymyxin B and nafamostat mesilate on endotoxaemia in conscious Guinea-pigs Clin Exp Pharmacol Physiol 2008 35 17 22 10.1111/j.1440-1681.2007.04741.x 18047622

[B33] Iba T Nagaoka I Yamada A Nagayama M Miki T Effect of hemoperfusion using polymyxin B-immobilized fibers on acute lung injury in a rat sepsis model Int J Med Sci 2014 11 255 261 10.7150/ijms.6276 24516349 PMC3917114

[B34] Aoki H Kodama M Tani T Hanasawa K Treatment of sepsis by extracorporeal elimination of endotoxin using polymyxin B-immobilized fiber Am J Surg 1994 167 412 417 10.1016/0002-9610(94)90126-0 8179086

[B35] Tani T Hanasawa K Endo Y Yoshioka T Kodama M Kaneko M Uchiyama Y Akizawa T Takahasi K Sugai K Therapeutic apheresis for septic patients with organ dysfunction: hemoperfusion using a polymyxin B immobilized column Artificial Organs 1998 22 1038 1044 10.1046/j.1525-1594.1998.06086.x 9876096

[B36] Zhou F Peng Z Murugan R Kellum JA Blood purification and mortality in sepsis: a meta-analysis of randomized trials Crit Care Med 2013 41 2209 2220 10.1097/CCM.0b013e31828cf412 23860248 PMC3758418

[B37] Cruz D Perazella M Bellomo R De Cal M Polanco N Corradi V Lentini P Nalesso F Ueno T Ranieri V Ronco C Effectiveness of polymyxin B-immobilized fiber column in sepsis: a systematic review Crit Care 2007 11 R47 10.1186/cc5780 17448226 PMC2206475

[B38] Vincent J Laterre P Cohen J Burchardi H Bruining H Lerma F Wittebole X De Backer D Brett S Marzo D Nakamura H John S A pilot-controlled study of a polymyxin B-immobilized hemoperfusion cartridge in patients with severe sepsis secondary to intra-abdominal infection Shock 2005 23 400 405 10.1097/01.shk.0000159930.87737.8a 15834304

